# Supplementary material for: Saccharomyces boulardii (CNCM I-745) improves intestinal damage in sepsis by remodeling intestinal flora structure
Source: Front Cell Infect Microbiol. 2025 Jul 31;15:1602792. doi: 10.3389/fcimb.2025.1602792 (PMC12350329; doi:10.3389/fcimb.2025.1602792)
Supplement: Supplementary file 1 [file DataSheet1.zip › summary/summary/6_Taxonomy_community/1_Taxonomy_profiling/7_Species/Group/Species_abund_top30.Group.stacked_bar.pdf]

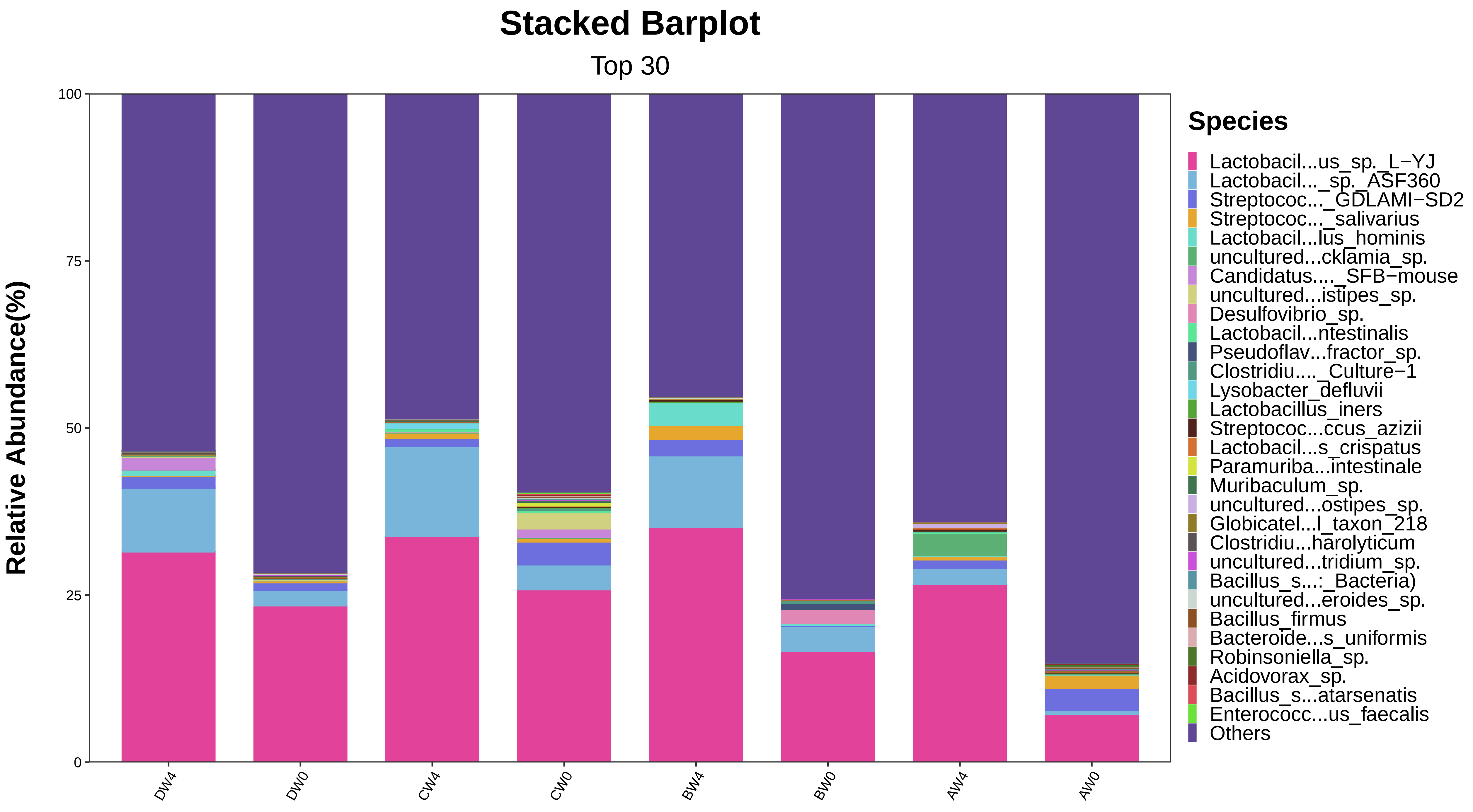
Stacked Barplot
Top 30
Relative Abundance(%)
100
75
50
25
0
DW4
DW0
CW4
CW0
BW4
BW0
AW4
AW0
Species
Lactobacil...us_sp._L−YJ
Lactobacil..._sp._ASF360
Streptococ..._GDLAMI−SD2
Streptococ..._salivarius
Lactobacil...lus_hominis
uncultured...cklamia_sp.
Candidatus...._SFB−mouse
uncultured...istipes_sp.
Desulfovibrio_sp.
Lactobacil...ntestinalis
Pseudoflav...fractor_sp.
Clostridiu...._Culture−1
Lysobacter_defluvii
Lactobacillus_iners
Streptococ...ccus_azizii
Lactobacil...s_crispatus
Paramuriba...intestinale
Muribaculum_sp.
uncultured...ostipes_sp.
Globicatel...l_taxon_218
Clostridiu...harolyticum
uncultured...tridium_sp.
Bacillus_s...:_Bacteria)
uncultured...eroides_sp.
Bacillus_firmus
Bacteroide...s_uniformis
Robinsoniella_sp.
Acidovorax_sp.
Bacillus_s...atarsenatis
Enterococc...us_faecalis
Others
